# Composition and Protein Precipitation Capacity of Condensed Tannins in Purple Prairie Clover (*Dalea purpurea Vent*.)

**DOI:** 10.3389/fpls.2021.715282

**Published:** 2021-09-28

**Authors:** Qianqian Huang, Tianming Hu, Zhongjun Xu, Long Jin, Tim A. McAllister, Surya Acharya, Wayne E. Zeller, Irene Mueller-Harvey, Yuxi Wang

**Affiliations:** ^1^College of Animal Science and Technology, Yangzhou University, Yangzhou, China; ^2^Agriculture and Agri-Food Canada, Lethbridge Research and Development Centre, Lethbridge, AB, Canada; ^3^College of Animal Science and Technology, Northwest A&F University, Xianyang, China; ^4^United States Department of Agriculture - Agricultural Research Service, U.S. Dairy Forage Research Center, Madison, WI, United States; ^5^School of Agriculture, Policy and Development, University of Reading, Reading, United Kingdom

**Keywords:** purple prairie clover, condensed tannin, botanical distribution, chemical structure, protein-precipitation capacity

## Abstract

This study aimed to determine the concentration and composition of condensed tannins (CT) in different tissues of purple prairie clover (PPC; *Dalea purpurea* Vent.) at different maturities and to determine their protein-precipitating capacity. The compositions of CT were elucidated after thiolysis with benzyl mercaptan followed by high-performance liquid-chromatography (HPLC) and ^1^H–^13^C heteronuclear single quantum coherence (HSQC) NMR spectroscopy. The results indicated that PPC flowering heads contained the highest CT concentration. Purple prairie clover CT consisted mainly of epicatechin (EC) and epigallocatechin (EGC) subunits. CT in the leaves were composed of more EC and less EGC than CT in stems and flowering heads at both the early flowering (EF) and late flowering (LF) head stages. The mean degree of polymerization was the highest for CT in stems and increased with maturity. CT isolated from PPC leaves at the early flowering head stage exhibited the greatest biological activity in terms of protein precipitation. Overall, the CT in PPC were predominantly procyanidins and the concentration and composition varied among the plant tissues and with maturity.

## Introduction

Condensed tannins (CT) are a class of secondary plant metabolites that are found in several forage plants. They possess various antimicrobial, anti-parasitic, anti-oxidant, and anti-inflammatory activities and as a result are seen as a promising natural alternative to in-feed antibiotics (Huang et al., 2017). CT can be beneficial or detrimental to ruminants, depending on the amount consumed and their chemical composition (Mueller-Harvey et al., 2019). The most widespread CT are oligomers and polymers of flavan-3-ol units composed of catechin (C), epicatechin (EC), gallocatechin (GC), or epigallocatechin (EGC) linked by carbon–carbon bonds (Hagerman and Butler, 1981). They are classified as procyanidins (PC) if derived from C and EC and as prodelphinidins (PD) if derived from GC or EGC. CT in different plants show large structural variation in terms of their mean degree of polymerization (mDP), proportions of PD, PC, and *cis*- or *trans* flavan-3-ol units (Ropiak et al., 2016).

Purple prairie clover (PPC; *Dalea purpurea* Vent.) is a native legume widely distributed across the North American prairie that contains a high concentration [up to 94 g/kg dry matter (DM)] of CT. The previous studies have shown that the PPC CT had mild effects on *in vitro* ruminal fermentation and that it was a highly palatable and nutritive prairie forage for ruminants, despite its high CT content (Jin et al., 2012; Huang et al., 2015; Peng et al., 2016). It has been shown that PPC CT exhibit strong anti-*Escherichia coli* O157:H7 activity in *in vitro* and *in vivo* studies (Liu et al., 2013; Wang et al., 2013; Huang et al., 2015; Jin et al., 2015). These findings indicate that the structural composition of PPC CT may play a key role in determining their biological activity. However, the composition of PPC CT is unknown. It has also been shown that the concentration and composition of CT in plants are affected by growing conditions, accessions, phenological growth stage, and tissue type (Theodoridou et al., 2011; Azuhnwi et al., 2013). The objectives of this study were to determine the concentration and composition of CT in different tissues of PPC at differing maturities and to determine their protein-precipitating capacity.

## Materials and Methods

### Preparation of PPC Plant Material

Whole PPC plants were harvested at vegetative (VEG; early June), early flowering heads (EF; middle July), and late flowering heads (LF; late August) stages from the three irrigated plots at Lethbridge Research and Development Center, Lethbridge, AB, Canada (latitude 49° 42′N, longitude 112° 45′W, and 911 m elevation). The plots were established under irrigation on the Swinton silt soil (Ayres et al., 1985). The plots were established by drilling seed into weed-free cultivated and packed soil treated with 100 kg of 34-17-0 fertilizer/ha. To control weeds during growing season, the plots were sprayed with Odyssey at the recommended rate of 42 g/ha with 500 ml of Merge in 20-gallon water. Further, 5 kg harvested forage from each individual plot was immediately manually separated into leaf, stem, and flowering head fractions and lyophilized. The fractions were ground to pass a 1.0 mm screen using a Thomas Wiley Cutting Mill (Arthur H. Thomas Co., Philadelphia, PA, USA) and stored in plastic bags in the dark at room temperature for the determination of CT concentration and *in situ* thiolysis of CT. Subsamples of each separate plant tissue were composited across the three plots to form a single sample for CT extraction and estimation of protein precipitating capacity.

### Determination of CT Concentration and CT Composition

The concentration of extractable CT was determined by the HCl-butanol method as previously described (Terrill et al., 1992) with CT purified from whole PPC plant used as a standard.

The *in situ* thiolysis method was used to determine the composition of CT directly in the PPC plant materials according to Fryganas et al. (2018) (as shown in Supplementary Section). This procedure cleaved the CT with benzyl mercaptan into their flavan-3-ol subunits as described in detail by Gea et al. (2011). The thiolytic reaction products were identified by their UV spectra and as their molecular chloride adducts (M + Cl)^−^ by liquid chromatography–mass spectrometry (LC-MS) analysis (as shown in Supplementary Table 1) (Gea et al., 2011; Fryganas et al., 2018). They were quantified based on the peak areas at 280 nm using published flavan-3-ol response factors against taxifolin (as shown in Supplementary Table 1) (Gea et al., 2011). This yielded data on the mean degree of polymerization (mDP) of the CT, molar percentages of PC (C + EC), and PD (GC + EGC), and molar percentages of *cis*- (EC + EGC) and *trans*- (C + GC) flavan-3-ols within the CT as described by Gea et al. (2011).

### Isolation and Purification of CT for Composition Analysis by NMR

Subsamples of each plant tissue described under the preparation of PPC plant material section above were subjected to the standard extraction with an acetone/water mixture. Briefly, approximately 10 g of each tissue sample (stems, leaves, and flowers) from the EF stage were extracted three times each with acetone/water (7:3, 100 ml each time) and the combined extracts from each sample were concentrated on a rotary evaporator to remove the acetone, extracted with dichloromethane (2 × 50 ml) to remove the non-polar components and the resulting aqueous layer was freeze dried. Portions of each of these samples were subjected to NMR analysis. The remaining portions of these extracts were then subjected to the batch purification technique (Method 4) using Sephadex LH-20 following the procedures described in Brown et al. (2017) and fractionated according to the following eluent scheme: 1:1 MeOH/H_2_O (Fraction 0, F0); 3:7 acetone/H_2_O (Fraction 1, F1); 1:1 acetone/H_2_O (Fraction 2, F2); 7:3 acetone/H_2_O (Fraction 3, F3); and 9:1 acetone/H_2_O (Fraction 4, F4). All the fractions were concentrated under reduced pressure via rotary evaporation to remove the volatile organic solvent, the resulting aqueous layers were freeze dried and samples of these fractions were subjected to NMR analysis.

### NMR Spectroscopy

NMR spectra of the tissue extracts and the purified CT fractions were obtained on an Avance 360 (^1^H 360.13 and ^13^C 90.55 MHz) instrument equipped with XWINNMR software (Bruker Corporation, Billerica, MA, USA) or on a BrukerBiospin DMX-500 (^1^H 500.13 and ^13^C 125.76 MHz) instrument equipped with Topspin 3.4 software (Bruker Corporation, Billerica, MA, USA). The ratios of procyanidin/prodelphinidin (PC/PD) and cis/trans stereochemistry and mDP were determined through integration of respective cross peak signals in the ^1^H–^13^C heteronuclear single quantum coherence (HSQC) NMR spectra according to the procedures previously reported (Zeller et al., 2015a; Fryganas et al., 2018; Naumann et al., 2018).

### Determination of the Protein–Precipitating Capacities of PPC CT

Condensed tannins from stem, leaf, and flowering heads of PPC harvested at VEG, EF, and LF stages were extracted and purified as previously described (Wang et al., 2009). The protein precipitation capacities of these purified CT were determined using a modified procedure described by McAllister et al. (2005). Bovine serum albumin (BSA) and Rubisco (MW 557 kDa) from spinach (Sigma-Aldrich, MO, USA) were used as model proteins for determining the relative capacities of extracted CT to bind protein. The BSA was dissolved (3 mg/ml) in 0.2 M acetate buffer (pH = 5.0) containing 0.17 M NaCl, and the Rubisco was dissolved (4 mg/ml) in 1 M 2-amino-2-(hydroxymethyl)-1, 3-propanediol hydrochloride (Tris HCl; pH = 7.8). Then, 1 ml of each protein solution was combined with 0.5 ml of aqueous solutions containing 0, 50, 100, 200, 300, 400, 500, 750, 1,000, 1,250, or 1,500 μg of CT from each source. Each mixture was vortexed, allowed to stand at room temperature for 30 min and centrifuged (15,600 × *g*, 10 min). The CT remaining in a 1-ml subsample of supernatant were removed by adding 0.5 ml of aqueous polyethylene glycol (Sigma-Aldrich; MW 6000; 12 mg/ml) followed by centrifugation (15,600 × *g*, 10 min). Protein remaining in solution was quantified colorimetrically (OD595) using a Dye Reagent Concentrate Kit (BioRad Laboratories, Mississauga, ON, Canada) against a freshly prepared solution of BSA or Rubisco protein as a standard. Each assay consisted of six replicates for each source and concentration of CT and the assay was repeated three times over a 1-week period.

The amount of protein precipitated was calculated as the difference between added protein and that present in the supernatant after CT addition. Data were fitted to a sigmoidal curve using non-linear regression in SigmaPlot for Windows (version 13.0; Systat Software Inc., Santa Jose, CA, USA):


$$\begin{array}{l} y\;=\;a0\;+\;a/\left(1\;+\;exp\left(-\left(x-b\right)/c\right)\right) \end{array}$$

where *y* = mg of protein (BSA or Rubisco) precipitated, *x* = μg of CT incubated, *a0* + *a* = estimated maximum amount (mg) of protein (BSA or Rubisco) precipitated, *b* = sigmoidal center (mg of CT at the 50% of maximal protein precipitation), and *c* = sigmoidal width. The protein-precipitating capacity of each CT was expressed as the amount (μg) of the CT required to precipitate 1.0 mg of BSA or Rubisco protein.

### Statistical Analysis

All data were analyzed using the MIXED procedure of SAS (SAS Institute Inc, 2012). Concentration data were analyzed by ANOVA with growth stage as a fixed effect and plot as a random factor. For flavan-3-ol units and structure composition, the data were originally analyzed using a growth stage × tissue type factorial arrangement. When the effects of growth stage × plant tissue interaction were significant, means were compared among plant tissues at each plant maturity. The growth stage and tissue type were treated as fixed effect individually and run was considered as a random factor with regards to the protein precipitating capacity of CT. The differences were determined using LSMEANS with the PDIFF option in SAS (SAS Institute, Inc., NC, USA) with significance declared at *p* < 0.05.

## Results

### Botanical Distribution of PPC CT

At all the growth stages, flowering heads contained the highest concentration of extractable CT, followed by leaves and stems (Table 1). The extractable CT concentration in stems of PPC was higher (*p* < 0.01) at VEG than the EF and LF, whereas it tended to be higher (*p* = 0.084) in flowering heads at EF than LF. There were no differences (*p* > 0.05) in CT content of leaves among the three stages.

**Table 1:** Concentration (grams per kilogram dry matter) of extractable condensed tannins (CT) in stems, leaves, and flowering heads of purple prairie clover (*Dalea purpurea* Vent) harvested at vegetative (VEG), early flowering heads (EF), or late flowering heads (LF).

	**VEG**	**EF**	**LF**	**SEM**	***p-*value**
Stems	46.1^a^	30.3^b^	22.4^b^	2.73	0.002
Leaves	74.8	63.9	60.8	5.75	0.27
Flowering heads	ND^1^	166	131	13.3	0.084

a,b *Means with different letters differ (p < 0.05)*.

1 *ND, not detected*.

### Flavan-3-ol Composition of PPC CT

**Figure 1:**
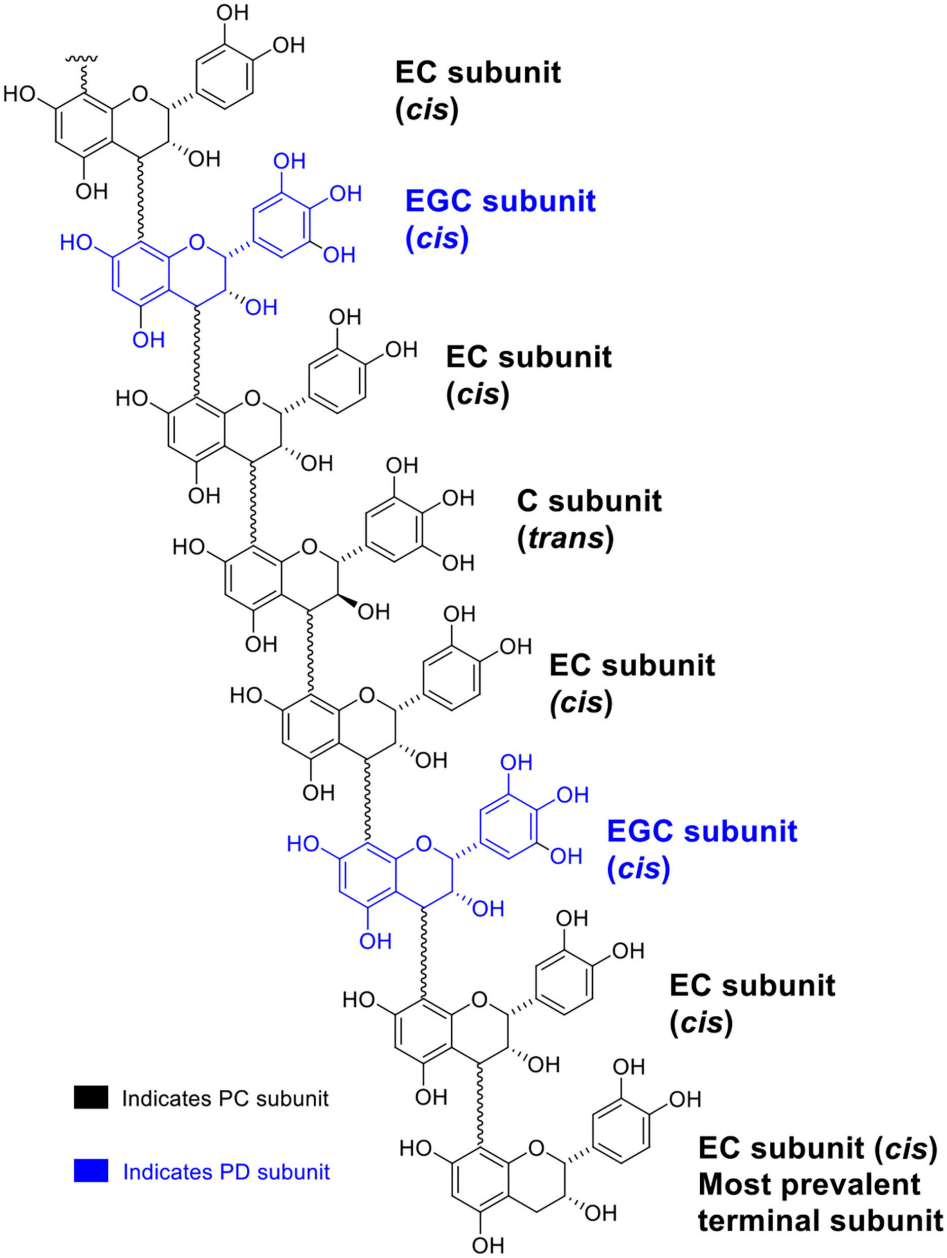
A generic structure illustrating the multitude of flavan-3-ol subunits of CT from PPC (*Dalea purpurea* Vent.).

**Figure 2:**
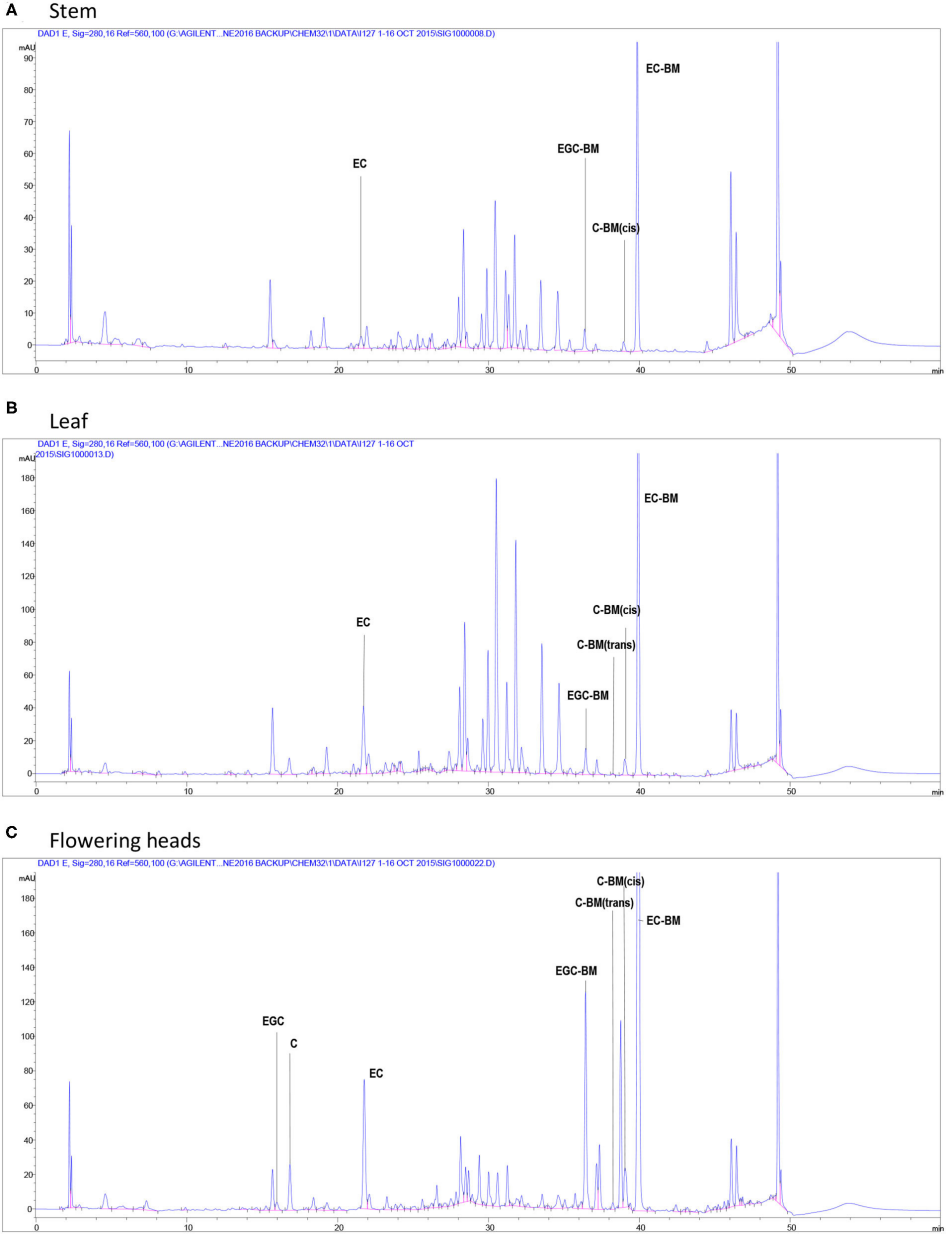
High-performance liquid chromatography chromatograms of flavan-3-ols after *in situ* thiolysis of CT in stems **(A)**, leaves **(B)**, at the vegetative stage and flowering heads **(C)** at the early flowering stage of PPC (*Dalea purpurea* Vent). Terminal units are released as a monomeric flavan-3-ols: EGC, epigallocatechin; C, catechin; EC, epicatechin; extension units are released as benzyl mercaptan (BM)-adducts: EGC-BM, 3,4-*trans*-epigallocatechin benzyl mercaptan; C-BM, 3,4-*cis*- or *trans*-catechin benzyl mercaptans; EC-BM, 3,4-*trans*-epicatechin benzyl mercaptan.

A generic structure of CT, consisting of the flavan-3-ol subunits found in PPC, is presented in Figure 1. Regardless of the plant tissues or maturity, PPC CT were mainly composed of EC (70–80%) and EGC (20–30%) subunits with GC not being detected (Table 2). The thiolysis reaction releases terminal units as C, EC, GC, EGC, and the extension units as their benzyl mercaptan derivatives (C-BM, EC-BM, GC-BM, and EGC-BM; Figure 2). On average ~85–97% of flavan-3-ols were associated with extension units, of which EC accounted for ~60–70% and EGC for ~15–30%. Gallocatechin, EGC, and C were not found in terminal units of stem or leaf CT at any growth stage and GC was also absent from flowering CT at EF and LF stages. CT in leaves, stems and flowering heads also did not contain GC in the extension units at any of the growth stages.

**Table 2:** Flavan-3-ol composition (mole %) of CT in stems, leaves, and flowering heads of purple prairie clover (PPC) (*Dalea purpurea* Vent) harvested at VEG, EF, or LF.

	**Terminal units (%)**				**Extension units (%)**			
	**GC**	**EGC**	**C**	**EC**	**GC**	**EGC**	**C**	**EC**
**VEG**								
Stems	0	0	0	3.83^b^	0	20.6	2.63^b^	72.9
Leaves	0	0	0	12.4^a^	0	16.1	3.77^a^	67.7
SEM	–	–	–	0.259	–	1.20	0.199	1.11
*p*-value	–	–	–	0.001	–	0.058	0.016	0.052
**EF**								
Stems	0	0^b^	0^b^	3.17^c^	0	30.1^a^	0.43^c^	66.3^a^
Leaves	0	0^b^	0^b^	14.7^a^	0	14.9^b^	4.80^a^	65.6^a^
Flowering heads	0	1.57^a^	1.47^a^	5.27^b^	0	28.0^a^	2.23^b^	61.4^b^
SEM	–	0.051	0.069	0.445	–	2.28	0.253	2.05
*p*-value	–	<0.001	<0.001	<0.001	–	0.001	<0.001	0.021
**LF**								
Stems	0	0^b^	0^b^	1.57^c^	0	35.3^a^	0.57^c^	62.6^b^
Leaves	0	0^b^	0^b^	9.97^a^	0	13.7^b^	6.38^a^	70.7^a^
Flowering heads	0	1.90^a^	1.23^a^	3.87^b^	0	35.0^a^	2.17^b^	55.8^c^
SEM	–	0.744	0.093	0.685	–	3.26	0.464	2.47
*p*-value	–	0.029	0.020	0.003	–	0.014	0.003	0.005

a,b,c *Means within a column with different letters differ (p < 0.05)*.

*GC, gallocatechin; EGC, epigallocatechin; C, catechin; EC, epicatechin*.

Condensed tannins in leaves of PPC harvested at VEG had greater proportion of EC in terminal units (*p* < 0.01) and C in the extension units (*p* < 0.05) than the stems, with no differences in the occurrence of EC and EGC in the extension units. Proportions of EC in terminal units and C in the extension units were in the order of leaf CT > flowering head CT > stem CT at EF (*p* < 0.001) and LF (*p* < 0.01) growth stages, respectively. However, the proportion of EGC in the extension units in leaf CT was much lower than in stem and flowering head CT at EF (*p* < 0.01) and LF (*p* < 0.05). The extension units in leaf and stem CT at EF contained a similar percentage of EC and were slightly higher than flowering head CT; while the percentage of EC in the extension units in leaf CT at LF was the highest, followed by those in stems and flowering heads (*p* < 0.01), respectively.

The terminal units in stem CT contained a higher (*p* < 0.05) percentage of EC at VEG and EF than that at LF, whereas the extension units in CT from stems contained a higher (*p* < 0.01) percentage of EC at VEG than at EF and LF. Similarly, the percentage of C in extension units in stem CT at VEG was higher (*p* < 0.01) than that at EF and LF. However, the percentage of EGC in the extension units in stem CT at VEG was lower (*p* < 0.01) than that at EF and LF. The proportion of EC in terminal units in leaf CT was highest at EF, followed by VEG and LF (*p* < 0.01). The percentage of EC in extension units in leaf CT at EF was lower (*p* < 0.05) than that at LF. CT in leaves contained the lowest proportion of C in extension units at VEG, followed by EF and LF (*p* < 0.001), respectively. Similar to the CT in stems and leaves, CT in flowering heads also contained a higher (p < 0.01) proportion of EC in terminal units at EF than that at LF. The proportions of EC, C, and EGC in extension units in flowering head CT at EF were similar to that at LF.

### Compositional Aspects of PPC CT

The mDP was the highest for stem CT and the lowest for leaf CT at all stages (*p* < 0.001, Table 3). The mDP of leaf CT at EF was the lowest, followed by VEG and LF (*p* < 0.001), respectively. Stem CT also had the highest mDP at LF (*p* < 0.01) but was similar at VEG and EF. CT in PPC were predominately PC regardless of tissue or growth stage. The percentage of PC tannins was higher (*p* < 0.050) in leaf CT than stem CT at all the growth stages. There were no differences in the percentage of PC tannins among different growth stages in either leaves or flowering heads. Stem CT at VEG had a greater (*p* < 0.01) percentage of PC than at EF and LF. The percentage of *cis* flavan-3-ol subunits was the highest in stem CT at all growth stages, followed by flowering head and leaf CT (*p* < 0.05). In contrast, the percentage of *cis* units of CT from different tissues was inconsistent across the growth stages. Stem CT had a greater (*p* < 0.01) proportion of *cis* units at EF and LF than that at VEG, whereas leaf CT had the greatest proportion of *cis* units at VEG, followed by EF and LF (*p* < 0.001), respectively.

**Table 3:** Composition of CT in stems, leaves, and flowering heads of PPC (*Dalea purpurea* Vent) harvested at VEG, EF, or LF.

	**mDP^1^**	**PC (%)**	**PD (%)**	***Cis* (%)**	***Trans* (%)**
**VEG**					
Stems	27.5	79.4	20.6	97.4	2.6
Leaves	8.1	83.9	16.1	96.2	3.8
SEM	0.31	1.19	1.19	0.21	0.21
*p*-value	<0.001	0.05	0.05	0.02	0.02
**EF**					
Stems	31.8^a^	69.9^b^	30.1^a^	99.6^a^	0.4^c^
Leaves	6.8^c^	85.1^a^	14.9^b^	95.2^c^	4.8^a^
Flowering heads	12.1^b^	70.4^b^	29.6^a^	96.3^b^	3.7^b^
SEM	1.12	2.30	2.30	0.26	0.26
*p*-value	<0.001	<0.001	<0.001	<0.001	<0.001
**LF**					
Stems	44.8^a^	64.7^b^	35.3^a^	99.4^a^	0.6^c^
Leaves	10.2^c^	84.8^a^	15.2^b^	93.9^c^	6.1^a^
Flowering heads	14.8^b^	63.0^b^	37.0^a^	96.6^b^	3.4^b^
SEM	2.14	2.97	2.97	0.36	0.36
*P-*value	<0.001	0.004	0.004	<0.001	<0.001

1 *mDP, mean degree of polymerization; PC (procyanidin), catechin (C) + epicatechin (EC); PD (prodelphinidin), gallocatechin (GC) + epigallocatechin (EGC); Cis, EC + EGC; Trans, C + GC*.

a,b,c *Means within a column with different letters differ (p < 0.05)*.

### NMR Spectroscopy Evaluation of CT Composition

**Figure 3:**
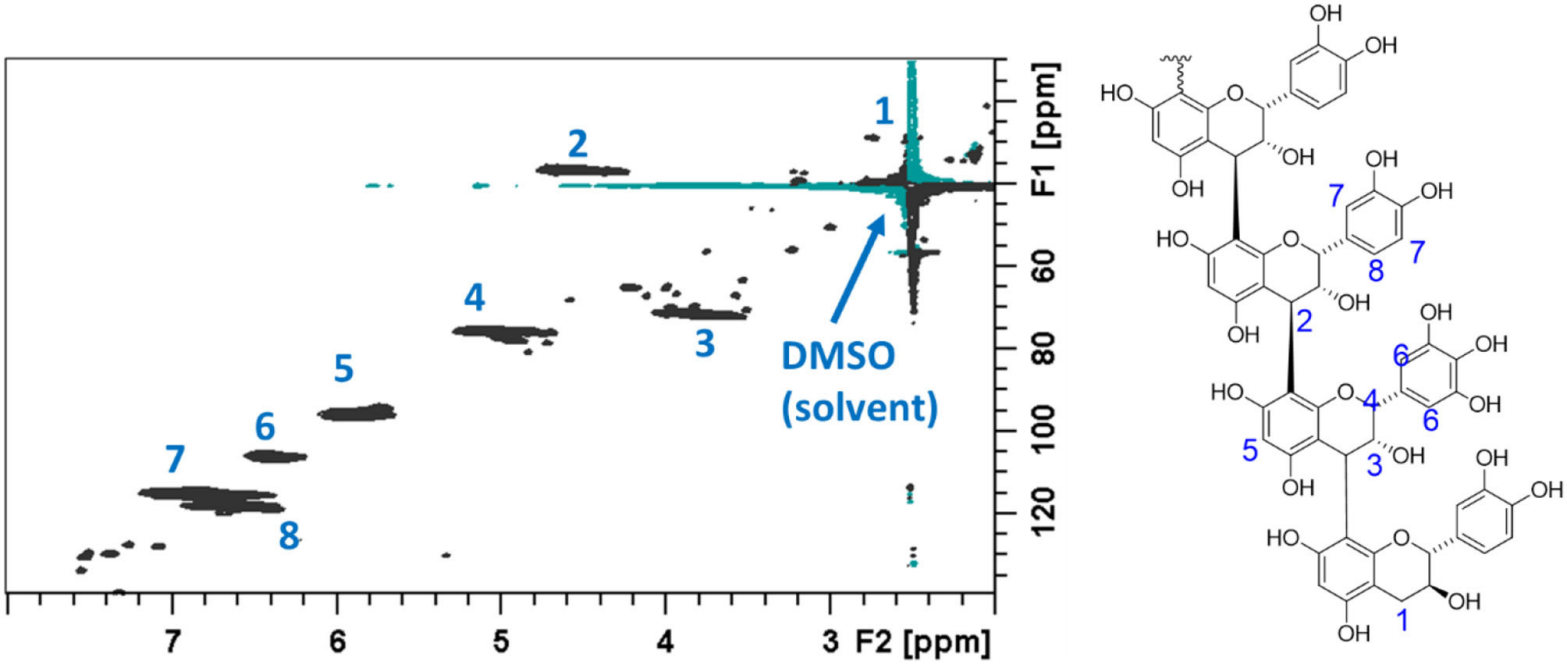
^1^H-^13^C heteronuclear single quantum coherence (HSQC) NMR spectrum of purified CT from flowering heads of PPC (*Dalea purpurea* Vent). The C–H bond cross peak signals arising from the condensed tannins present in this sample are indicated on the spectrum and on the accompanied partial CT structure.

The NMR spectra obtained from the acetone/water extracts and the fractions from the CT purifications were evaluated for CT composition according to the previous reports (Zeller et al., 2015a; Naumann et al., 2018). For the sake of completeness, detailed explanations of CT compositional analysis by this 2D NMR technique are included in the Supplemental Material. The results from these evaluations are provided in Table 4, with comparison values from the corresponding EF samples analyzed by *in situ* thiolysis. A ^1^H–^13^C HSQC NMR spectrum of purified CT from the flowering heads, identifying the cross peaks due to CT signals, is given Figure 3.

**Table 4:** Comparison of thiolysis and NMR data for the composition of CT in stems, leaves, and flowers of PPC (*Dalea purpurea* Vent) harvested at EF stage.

		**mDP^1^**	**PC (%)**	**PD (%)**	***Cis* (%)**	***Trans* (%)**
Stems	Thiolysis	31.8	69.9	30.1	99.6	0.43
NMR	F2	10.8 ± 0.3	82.4 ± 0.2	17.6 ± 0.2	92.2 ± 0.7	7.8 ± 0.7
	F3	17.2 ± 0.2	79.6 ± 1.0	20.4 ± 1.0	91.9 ± 1.6	8.1 ± 1.6
Leaves	Thiolysis	6.84	85.1	14.9	95.2	4.82
NMR	Extraction	6.45 ± 0.33	90.2 ± 1.1	9.8 ± 1.1	94.9 ± 0.1	5.1 ± 0.1
	F2	11.1 ± 0.1	90.6 ± 0.2	9.4 ± 0.2	95.3 ± 0.2	4.7 ± 0.2
	F3	12.2 ± 1.1	90.2 ± 0.3	9.8 ± 0.3	93.5 ± 1.0	6.5 ± 1.0
Flowers	Thiolysis	12.1	70.4	29.6	96.3	3.70
NMR	Extraction	7.8 ± 0.2	71.2 ± 0.5	28.8 ± 0.5	95.2 ± 0.9	4.8 ± 0.9
	F2	11.6 ± 0.8	76.2 ± 0.9	23.8 ± 0.9	87.4 ± 2.6	12.6 ± 2.6
	F3	ND	72.5 ± 1.1	27.5 ± 1.1	96.0 ± 0.2	4.0 ± 0.2

1 *mDP, mean degree of polymerization; PC (procyanidin), catechin (C) + epicatechin (EC); PD (prodelphinidin), gallocatechin (GC) + epigallocatechin (EGC); Cis, EC + EGC; Trans, C + GC. ND, not determined; signal to noise ratio of the terminal H/C-4 cross peaks too low for accurate measurement*.

### Protein-Precipitating Capacities of CT in PPC

**Figure 4:**
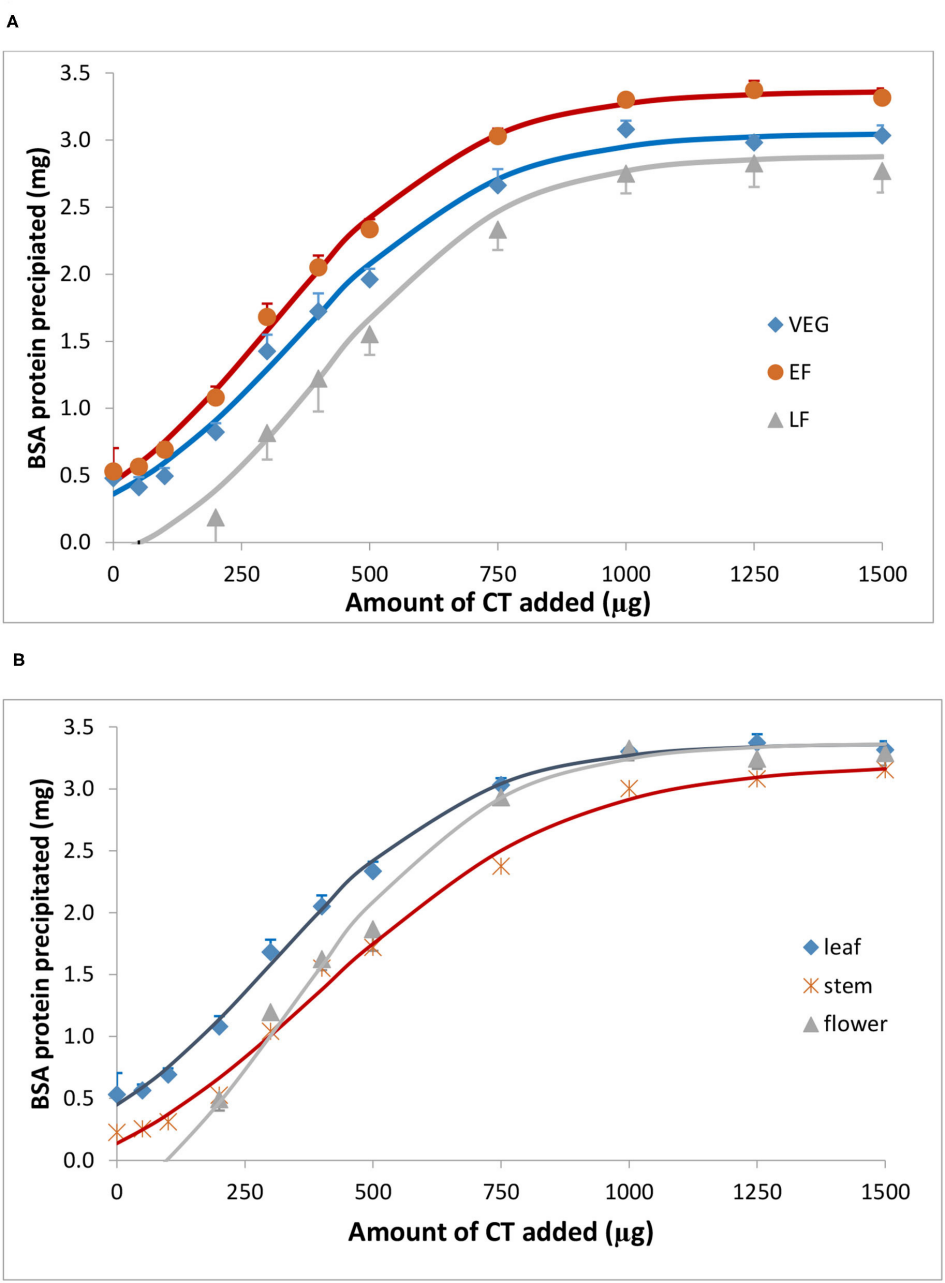
Precipitation of bovine serum albumin (BSA) by CT isolated from the leaves of PPC (*Dalea purpurea* Vent.) at VEG, EF, and LF stages **(A)**, and from leaves, stems, and flowering heads at early flowering heads **(B)**. Lines relate to corresponding CT data which were fitted to a SigmaPlot curve as described under statistical analysis. Bars indicate standard error and where not visible, fall within symbols.

**Figure 5:**
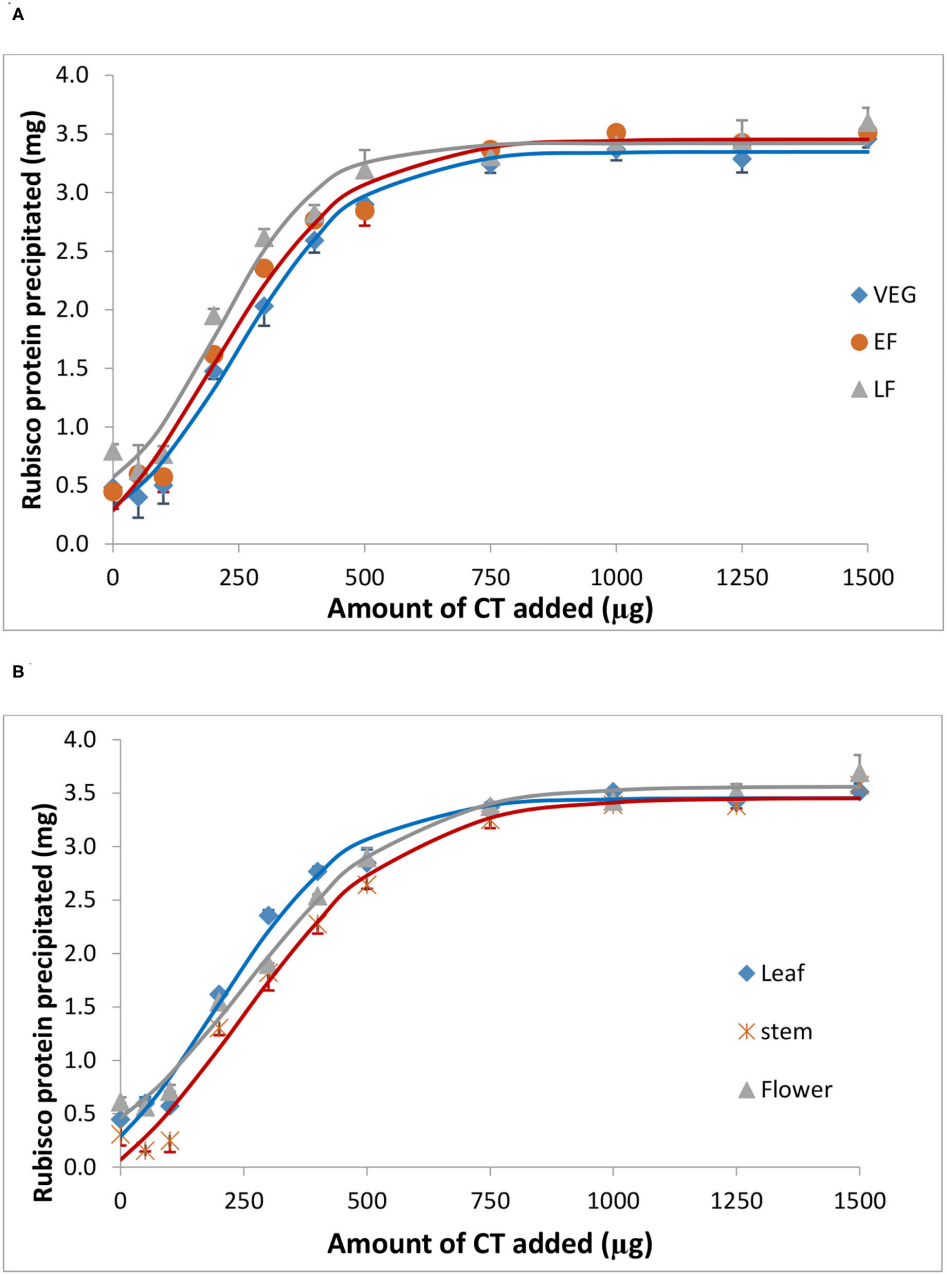
Precipitation of spinach ribulose 1,5-disphosphate carboxylase (Rubisco) by CT isolated from the leaves of PPC (*Dalea purpurea* Vent.) at VEG, EF, and LF growth stages **(A)**, and from leaves, stems, and flowering heads at the early flowering heads **(B)**. Lines relate to corresponding CT data which were fitted to a SigmaPlot curve as described under statistical analysis. Bars indicate SE and where not visible, fall within the symbols.

Bovine serum albumin was completely precipitated by ≥1,000 μg PPC CT (Figure 4), whereas Rubisco was completely precipitated by ≥ 750 μg PPC CT (Figure 5), irrespective of the growth stage or plant tissue. The protein-precipitating capacity of CT from the leaves of PPC harvested at EF was the highest, while it was the lowest for CT in PPC leaves harvested at VEG (*p* < 0.001) for BSA (Table 5). There were no differences (*p* > 0.05) in the ability of these fractions to precipitate Rubisco. CT in the leaves of PPC harvested at EF exhibited a greater (*p* < 0.001) ability to bind to BSA and Rubisco protein than CT obtained from stems or flowering heads (Table 6). The protein-precipitating capacity of CT from flowering heads at EF for BSA was similar to that of CT obtained from stems, whereas it was similar to that of CT obtained from leaves for Rubisco (*p* > 0.05).

**Table 5:** Parameters of precipitating capacities of CT from leaves of PPC (*Dalea purpurea* Vent) harvested at VEG, EF, or LF.

**Protein^1^**	**Parameters^2^**	**VEG**	**EF**	**LF**	**SEM**	***p*-value**
BSA	*a0 + a1*	3.0^b^	3.4^a^	2.9^b^	0.06	0.01
	*b*	349^a^	152^c^	202^b^	6.1	<0.001
	*c*	192^a^	97^b^	89^b^	5.9	<0.001
	*PP*	231^a^	84^c^	177^b^	2.7	<0.001
Rubisco	*a0 + a1*	3.3	3.4	3.4	0.07	0.559
	*b*	243	193	206	15.8	0.143
	*c*	122^ab^	138^a^	100^b^	7.8	0.039
	*PP*	148	122	94	16.3	0.141

a,b,c *Means within a row with different letters differ (p < 0.05)*.

1 *BSA, bovine serum albumin; Rubisco, ribulose 1,5-disphopshate carboxylase*.

2 *Parameters were obtained by fitting the amount of precipitated protein (mg) and amount of CT (μg) incubated with equation: y = a0 + a/(1 + exp(–(x–b)/c)), where y = mg of protein (BSA or Rubisco) precipitated, x = μg of CT incubated, a0+a = estimated maximal amount (mg) of protein (BSA or Rubisco) precipitated, b = sigmoidal center (μg of CT at the 50% of maximal protein precipitation), and c = sigmoidal width; PP, Protein-precipitating capacity; expressed as μg CT required to precipitate 1 mg of protein*.

**Table 6:** Parameters of precipitating capacities of CT from stems, leaves, and flowering heads of purple prairie clover (PPC) (*Dalea purpurea* Vent) harvested at EF.

**Protein**	**Parameters**	**Stem**	**leaf**	**flowering**	**SEM**	***p*-value**
BSA	*a0 + a1*	3.2	3.4	3.4	0.06	0.156
	*b*	199	152	166	22.3	0.317
	*c*	123	97	96	8.9	0.1256
	*PP*	149^a^	84^b^	145^a^	2.0	<0.001
Rubisco	*a0 + a1*	3.4	3.4	3.4	0.02	0.735
	*b*	240	193	220	21.8	0.366
	*c*	166	138	126	6.6	0.013
	*PP*	189^a^	122^b^	124^b^	10.4	0.016

a,b *Means within aa row with different letters differ (P < 0.05)*.

1 *BSA, bovine serum albumin; Rubisco, ribulose 1,5-disphopshate carboxylase*.

2 *Parameters were obtained by fitting the amount of precipitated protein (mg) and amount of CT (μg) incubated with equation: y = a0 + a/(1 + exp(–(x–b)/c)), where y = mg of protein (BSA or Rubisco) precipitated, x = μg of CT incubated, a0 + a = estimated maximal amount (mg) of protein (BSA or Rubisco) precipitated, b = sigmoidal center (μg of CT at the 50% of maximal protein precipitation), and c = sigmoidal width; PP, Protein-precipitating capacity; expressed as μg CT required to precipitate 1 mg of protein*.

## Discussion

### CT Concentrations in Different Plant Tissues at Different Phenological Stages

The concentration of CT in plants is tissue-specific (SAS Institute Inc, 2012), with the highest CT concentrations in forages such as birdsfoot trefoil (*Lotus corniculatus*) and white prairie clover (*Dalea candida*) being associated with flowers (Terrill et al., 1992; Gebrehiwot et al., 2002; Li et al., 2014). However, in sainfoin (*Onobrychis viciifolia*), CT are most abundant in the leaves (Guglielmelli et al., 2011; Li et al., 2014). The tissue distribution of CT in PPC as revealed by this study was consistent with the previous reports (Jin et al., 2012; Li et al., 2014). The reason why CT are concentrated in flowers is not fully understood. It might represent a defensive strategy for the reproductive tissues of plants, protecting from being eaten by the insects during pollination (Barry, 1989). Meanwhile, polyphenols in the flowers aid in attracting the pollinating insects to help ensure that the fertile seeds are produced (Li et al., 2014).

Phenological development is considered as one of the factors that can affect the CT content in plants (Frutos et al., 2004). The studies (Berard et al., 2011; Li et al., 2014) screened the CT concentration of prairie legume forages in different phenological stages in western Canada and found that the CT content in all species was higher at the flowering than at the vegetative stage, with the exception of sainfoin which had higher CT content at the vegetative stage. Similarly, this study showed that the CT concentration in whole PPC plants increased with advancing maturity, a finding consistent with our previous study (Jin et al., 2012). This pattern reflects the increase in the proportion of flowering heads which have the highest CT content within the whole plant. This response could be partly explained by the growth-differentiation balance hypothesis; whereby the plants utilize more available resources for producing biomass in the vegetative stage leaving few resources for the synthesis of secondary metabolites, such as tannins (Cella Pizarro and Bisigato, 2010). However, at the flowering stage, excess carbon may be available for the synthesis of secondary metabolites, such as CT. The previous studies also showed a decline in the CT concentration in leaves from PPC, white prairie clover, and sainfoin as they advanced from vegetative to the flowering stages (Jin et al., 2012; Li et al., 2014). In this study, a significant decline in CT concentration was observed in stems during maturation from vegetative to flowering head stages with no differences between the leaf and flowering fractions. Indeed, it has been suggested that CT in roundhead lespedeza may be translocated from the stems and leaves to the flowering heads (Springer et al., 2002). Alternatively, it has also been speculated that CT in sericea lespedeza (*Lespedeza cuneata*) might be involved in the photosynthetic transport and in other physiological processes within the plant (Mosjidis et al., 1990).

### Flavan-3-ol Composition of PPC CT

The polymers of PPC CT mainly consist of EC, C, and EGC and no GC was detected. Epicatechin (~72.1%) and EGC (~24.6%) were the dominant flavanols in terminal and extension units. Thus, PPC CT have a high procyanidin: Prodelphinidin (PC:PD) ratio (~75:25) and *cis:trans* ratio (~96.8:3.2). These results agree with our previous study with PPC (Peng et al., 2018) and are corroborated through the results obtained by NMR spectroscopy on the CT extract and purified CT fractions from the EF stem, leaf, and flower purifications (Table 4). Even though thiolysis was conducted directly on the plant material and NMR analyses were conducted on the extracted and then, purified CT samples, the data in Table 4 is remarkably similar with a few exceptions. For analysis of the EF leaves, the NMR data from the initial extraction and the thiolysis data are extremely consistent, with mDP and the PC/PD and *cis*/*trans* ratios remarkably close in value. The PC/PD and *cis*/*trans* ratios from both thiolysis and NMR data from the flower extract are remarkably similar with only the mDP from these two diverse methods differing substantially. Although somewhat removed from the measurement of CT composition directly from plants, such as in the *in situ* thiolysis conducted here, NMR examination of purified fractions shows that the pertinent compositional information can be obtained from examination of this data. However, loss of both low and high molecular weight CT during this fractionation process can lead to selected fraction compositions varying somewhat from the entire CT profile (Brown et al., 2017). Unfortunately, the ^1^H–^13^C HSQC NMR spectrum of the acetone/water extract from stems was not usable for composition analysis due to the host of interfering cross peaks signals from the impurities present in the extract (Supplementary Figure 1). Thus, the NMR analysis for CT from EF stems were performed on F2 and F3 of the CT purification conducted on Sephadex LH-20. High molecular weights CT are retained on the Sephadex LH-20 resin and thus the mDP determinations from these fractions on the samples containing a high mDP tend to be lower than whole plant determinations, accounting for the lower observed mDP from fractions vs. the whole tissue thiolytic analysis. This fractionation can result in varied PC/PD and *cis*/*trans* ratios (F2 and F3 of stem determination) but can also reaffirm the compositional determinations of these techniques (F2 and F3 of leaves and flowers). Overall, the results from thiolysis and NMR analyses were shown to be highly corroborative in nature and these results confirm that *in situ* thiolysis is a robust, reliable, and straight-forward method for analyzing the CT in plant materials directly.

This composition of CT in PPC is similar to that of CT in *Lotus corniculatus*, which also has predominantly PC-type units with EC dominating and the absence of GC (Foo et al., 1996). Conversely, CT from some other common forages, such as sainfoin (Koupai-Abyazani et al., 1993) and *L. pedunculatus* (Foo et al., 1997) contain a predominance of PD-type subunits with EGC being the prevalent monomer and the presence of GC. Similar to this study, sainfoin CT also contain more *cis*- than *trans*-isomer units (Koupai-Abyazani et al., 1993). The mDP of PPC CT in this study is consistent with our earlier study, and is comparable with that of *L. pedunculatus* CT, but is larger than that of *L. corniculatus* CT (Peng et al., 2018). These structural variations of CT across different forages may contribute to their varying biological activities, which may further explain the observed differences in the nutritional effects of these forages on ruminants.

Consistent with the results of CT concentration, the composition of PPC CT was also tissue specific. In general, CT in PPC leaves contained more EC and less EGC units compared with that in the flowering heads and stems, resulting in a higher proportion of PC in leaves than flowering heads and stems. Moreover, C and EGC were only present as extension units in CT from stems and leaves of PPC, whereas they were both present as terminal and extension units in CT from the flowering heads. Although CT concentration in PPC stems was the lowest, the mDP of CT polymers in PPC stems was noticeably higher than that of CT in PPC flowering heads and leaves. The negative correlation between CT concentration and mDP was also observed in sainfoin, indicating that the stems contained less CT, but higher molecular weight polymers (Stringano et al., 2012). These differences also occurred between leaves and stems with regard to the composition of CT in sainfoin (Theodoridou et al., 2011). These researchers have found that the PD concentration and mDP in sainfoin were higher in leaves than in stems, but the occurrence of *cis* isomers was reduced.

The structural characteristics of the stem, flowering heads, and leaf CT of PPC differed among the phenological stages. The content of C and EC decreased while that of EGC increased in CT polymers with advancing maturity thus the percentage of PC units declined. The mDP of CT polymers in PPC stems increased as the plant matured and as result the CT concentration in stems declined. This finding is consistent with the hypothesis that there is a negative correlation between the concentration and mDP of CT (Stringano et al., 2012). The content of EC decreased significantly in the CT of PPC flowering heads at the LF stage that contributed to a decrease in PC and *cis* content. There was no significant change in the flavan-3-ol composition of CT in PPC leaves as the plant matured, but the mDP of CT polymers initially slightly decreased and then increased as the plant matured. This phenomenon was also observed in sainfoin leaves (Koupai-Abyazani et al., 1993). All these results indicated that CT polymerization is a dynamic process, and that polymer composition undergoes significant changes as the plant advances through its growth cycle.

### Protein-Precipitating Capacity of PPC CT

The affinity of CT for proteins is a significant characteristic of CT and is primarily responsible for their biological activity (Haslam, 1989). The protein binding ability of CT depends on their chemical traits, such as molecular size, PC: PD ratio, mDP, number of potential hydrogen and hydrophobic bonding sites, and conformation (Hagerman and Butler, 1981; Foo et al., 1996). Therefore, the variation of the protein-precipitating capacity of PPC CT in different phenological tissues of differing maturity might be related to the varying chemical composition of CT in this study. Currently, most of the studies demonstrated that the capacity of CT to precipitate proteins increases with increasing concentrations of mDP (Osborne and McNeill, 2001; Dobreva et al., 2012; Saminathan et al., 2014; Zeller et al., 2015b), PD (Jones et al., 1976; Mangan, 1988), and *cis*-flavanol units (De Freitas and Mateus, 2001). However, this study found that the protein-precipitating capacity of PPC CT was higher in the leaves than in the flowering heads and stems, even though mDP, the percentage of PD and *cis*-flavanol units in leaf CT was lower than in the stem and flowering head CT. The same phenomenon was also observed for leaf CT at different phenological stages. It has been suggested that an mDP above eight would not further increase the affinity of CT for proteins (Harbertson et al., 2014). Similarly, it was also noticed that CT with an mDP of seven resulted in the saturation of their affinity for BSA (Ropiak et al., 2017). The mDP of CT from PPC leaves were 8.10, 6.84, and 10.2 at VEG, EF, and LF stages, respectively, which exceeded or were close to this saturation point, but these values averaged 34.4 and 13.45 for stem and flowering head CT, respectively. This may partially explain the observation that the CT isolated from EF leaves possessed the greatest protein-precipitation capacity in this study.

The concentration, structural composition, and protein-precipitating capacity of PPC CT varied with stage of maturity and among the leaves, stems, and flowering heads. Pure prairie clover flowering heads contained the highest CT concentration, followed by leaves and stems. Regardless of plant tissue or maturity, PPC CT consisted mostly of EC and EGC monomers. Purple prairie clover CT were predominantly of PC type which decreased as the plant matured. The mDP was highest for CT in stems and lowest for CT in leaves and the mDP increased as the plant matured. CT from leaves had a greater protein-precipitating capacity than CT from stems or flowering heads. CT isolated from leaves at EF exhibited the greatest protein-precipitation capacity. The different protein-precipitating capacities of PPC CT in the different tissues of differing maturity is likely a reflection of the chemical composition of CT.

## Data Availability Statement

The original contributions presented in the study are included in the article/Supplementary Material, further inquiries can be directed to the corresponding author/s.

## Author Contributions

YW conceived and designed the experiments. QH, LJ, and ZX performed the experiments. WZ and IM-H analyzed the structure information of CT. QH and YW analyzed the data. YW, TM, SA, and TH contributed reagents, materials, and analysis tools. YW, TM, and SA wrote the paper. All authors contributed to the article and approved the submitted version.

## Funding

This study was partially supported by Agriculture and Agri-Food Canada-Beef Science Cluster, Alberta Livestock and Meat Agency, and projects of Natural Science Foundation of Jiangsu Province of China (Grant No. BK20170494), National Natural Science Foundation of China (Grant No. 31902191), and China Postdoctoral Science Foundation (Grant No. 2017M611930). QH acknowledges the China Scholarship Council for awarding scholarship to conduct this research at Lethbridge Research and Development Center of Agriculture and Agri-Food Canada.

## Author Disclaimer

Mention of trade names or commercial products in this article is solely for the purpose of providing specific information and does not imply recommendation or endorsement by the U.S. Department of Agriculture (USDA). USDA is an equal opportunity provider and employer.

## Conflict of Interest

The authors declare that the research was conducted in the absence of any commercial or financial relationships that could be construed as a potential conflict of interest.

## Publisher's Note

All claims expressed in this article are solely those of the authors and do not necessarily represent those of their affiliated organizations, or those of the publisher, the editors and the reviewers. Any product that may be evaluated in this article, or claim that may be made by its manufacturer, is not guaranteed or endorsed by the publisher.

## Supplementary Material

Details on how the thiolysis degradation and LC-MS analysis were conducted and ^1^H-^13^C HSQC NMR spectral information are given in the Supplementary Material along with examples of ^1^H-^13^C HSQC NMR spectra used to conduct the composition analyses (stem, leaf, and flower extracts and purified fractions of CT from PPC flowers).

## Abbreviations

BM: benzyl mercaptan
BSA: bovine serum albumin
C: catechin
CT: condensed tannins
EC: epicatechin
EGC: epigallocatechin
EF: early flowering head stage
GC: gallocatechin
LF: late flowering head stage
mDP: mean degree of polymerization
PC: procyanidins
PD: prodelphinidins
PPC: purple prairie clover
VEG: vegetative growth stage.
