# Supplementary material for: Composition and Protein Precipitation Capacity of Condensed Tannins in Purple Prairie Clover (Dalea purpurea Vent.)
Source: Front Plant Sci. 2021 Sep 28;12:715282. doi: 10.3389/fpls.2021.715282 (PMC8509305; doi:10.3389/fpls.2021.715282)
Supplement: Supplementary file 1 [file Data_Sheet_1.docx]

**Supplemental Material for:**

Composition and protein precipitation capacity of condensed tannins in purple prairie clover (*Dalea purpurea Vent*.)

Qianqian Huang, Tianming Hu, Zhongjun Xu, Long Jin, Tim A. McAllister, Surya Acharya, Wayne E. Zeller, Irene Mueller-Harvey, Yuxi Wang

**Table of Contents**

Procedure for conducting in situ thiolysis………………………………………………..3

Liquid chromatography–mass spectrometry (LC–MS) analysis…………………………3

Table S1: List of *m/z* values of flavan-3-ols and their benzyl mercaptan

(BM) adducts detected as chloride adducts by LC-MS plus their molar

response factors relative to taxifolin (or dihydroquercetin) at 280 nm...............................4

Figure S1. Diagram showing the chemical structures of the benzyl mercaptan

adducts generated from thiolysis of PCC CT......................................................................5

Details of NMR Spectroscopy Acquisition……………...………………………………..5

Figure S2. ^1^H-^13^C HSQC NMR spectrum (500/125 MHz) of the

acetone/water extract isolated from stems of purple prairie clover

(*Dalea purpurea* Vent) at the EF stage..............................................................................6

Figure S3. ^1^H-^13^C HSQC NMR spectrum (500/125 MHz) of the

acetone/water extract isolated from leaves of purple prairie clover

(*Dalea purpurea* Vent) at the EF stage..............................................................................6

Figure S4. ^1^H-^13^C HSQC NMR spectrum (500/125 MHz) of the

acetone/water extract isolated from flowering heads of purple prairie clover

(*Dalea purpurea* Vent) at the EF stage..............................................................................7

Figure S5. ^1^H-^13^C HSQC NMR spectrum (360/90 MHz) of the F2 Fraction

(1:1 acetone/water) isolated from flowering heads of

purple prairie clover (*Dalea purpurea* Vent).....................................................................7

Figure S6. ^1^H-^13^C HSQC NMR spectrum (360/90 MHz) of the F3 Fraction

(7:3 acetone/water) isolated from flowering heads of

purple prairie clover (*Dalea purpurea* Vent).....................................................................8

Figure S7. ^1^H-^13^C HSQC NMR spectrum (500/125 MHz) of the F3 Fraction

(9:1 acetone/water) isolated from flowering heads of

purple prairie clover (*Dalea purpurea* Vent).....................................................................8

Calculating Procyanidin/Prodelphinidin (PC/PD) and

cis/trans-Flavan-3-ol Ratios, and mDP………………………………….………………9

Figure 8S. Diagram showing the NMR cross peaks integrated to provide

PC and PD content of CT fractions according to equation 1 above…………………...10

Figure 9S. Diagram showing the NMR cross peaks integrated to provide

*cis* and *trans* content of CT fractions according to equation 2 above…………………10

Figure 10S. Diagram showing the NMR cross peaks integrated to provide

a calculation of mDP of CT fractions. See discussion of

mDP determination given above………………………………………………………11

References……………………………………………………………………………..11

***In situ* thiolysis of condensed tannins directly in the PPC plant samples using a modification of the Gea *et al*. (2011) method as described by Fryganas *et al.* 2018:**

Note: We provide here the full thiolysis method details for the CT analysis in PPC samples, which were only slightly adapted from the publication of Fryganas, C., Drake, C., Ropiak, H.M., Mora-Ortiz, M., Smith, L.M.J., Mueller-Harvey, I., Kowalczyk, R.M. 2018. Carbon-13 Cross polarization magic angle spinning nuclear magnetic resonance for measuring proanthocyanidin content and procyanidin to prodelphinidin ratio in sainfoin (*Onobrychis viciifolia*) tissues. *J. Agric. Food Chem.* 66: 164073-4081.

Milled freeze-dried sainfoin material (200 mg) was weighed into a 10 mL screw-capped vial, dissolved in methanol (2 mL) and acidified with HCl (1 mL, 3.3% in methanol, v/v). This was followed by addition of benzyl mercaptan (100 µL) and the mixture was stirred in a water-bath for 1 h at 40 °C. The reaction was stopped by transferring the vials into an ice-bath and by adding 1 % formic acid in ultrapure water (9 mL) at room temperature. The samples were centrifuged (3000 rpm, 5 min) and 1 mL of the mixtures added to HPLC vials for LC-MS analysis. The reaction products were analyzed by LC–MS (see below). The concentrations of free flavan-3-ol monomers were also determined because they interfere with calculation of the mean degree of polymerization and CT composition (Gea et al., 2011).^16^ Free flavan-3-ols were measured directly in PPC plant samples by LC–MS using the above ‘thiolysis reagent’ and reaction conditions for the extraction, where the HCl-methanol (1 mL) and benzyl mercaptan (100 μL) reagents had been replaced with methanol (1100 μL).

**Liquid chromatography–mass spectrometry (LC–MS) analysis:**

Samples (5 µL) were injected into an HPLC Agilent 1100 series system (Agilent Technologies LDA, UK Limited, Stockport, UK) connected to an ACE super C_18_ column (5 μm; 150 x 3 mm; Hichrom Ltd; Theale; UK) fitted to an ACE guard column. The column temperature was set at 60 °C. The HPLC system consisted of a G1379A degasser, a G1312A binary pump, a G1313A ALS autoinjector, a G1314A VWD UV detector and a G1316A column oven and an API-ES instrument Hewlett Packard 1100 MSD Series (Agilent Technologies, Waldbronn, Germany). Data were acquired and processed with ChemStation software (version A 10.01 Rev. B.01.03). The flow rate was 0.4 mL/min using formic acid (1%) in water containing 100 mg/L ammonium chloride (solvent A) and HPLC-grade acetonitrile (solvent B). The thiolysis reaction products were eluted with the following gradient: 0-7 min, 2.5% B; 7-15 min, 2.5-5% B; 15-22 min, 5-10% B; 22-40 min, 10-40% B; 40-45 min, 40-100% B; 45-49 min, 100-2.5% B; 49-60 min, 2.5% B. Mass spectra were recorded in the negative ionization scan mode between *m/z* 100 and 1000. The mass spectrometer operating conditions were as follows: 3000 V for capillary voltage, nebulizer gas pressure at 35 psi, drying gas at 12 mL/min and dry heater temperature at 350 °C. Flavan-3-ols resulting from terminal subunits and their benzyl mercaptan adducts resulting from extension subunits were identified by their retention times, ultraviolet (UV) spectra (<https://pubs.acs.org/doi/suppl/10.1021/jf103609p/suppl_file/jf103609p_si_002.pdf>) and molecular masses as chloride adducts (M+Cl)^-^ (see Table below). They were quantified using peak areas at 280 nm and published response factors against taxifolin (see Table below).

This provided information on CT composition in terms of % terminal and % extension flavan-3-ol units (molar percentages); it also allowed calculation of the mean degree of polymerization (mDP), the percentage of procyanidins (PC) and prodelphinidins (PD), and of *cis*- and *trans*-flavan-3-ols in the CT (Gea *et al.*, 2011).^16^

**Table S1:** List of *m/z* values of flavan-3-ols and their benzyl mercaptan (BM) adducts detected as chloride adducts by LC-MS plus their molar response factors relative to taxifolin (or dihydroquercetin) at 280 nm (see Gea *et al* .*,* 2011).^16^

| **Flavan-3-ol and their abbreviations** | **Molecular ions as chloride adducts [M+Cl]^-^**  ***m/z*** | **Molar response factors at 280 nm** |
| --- | --- | --- |
| Gallocatechin (GC) | 341 | 0.06 |
| Epigallocatechin (EGC) | 341 | 0.06 |
| Catechin (C) | 325 | 0.30 |
| Epicatechin (EC) | 325 | 0.30 |
| 3,4-*trans*-gallocatechin-BM (*trans*-GC-BM) | 463 | 0.06 |
| 3,4-*cis*-gallocatechin-BM (*cis*-GC-BM) | 463 | 0.06 |
| 3,4-*trans*-epigallocatechin-BM (EGC-BM) | 463 | 0.06 |
| 3,4-*trans*-catechin-BM (*trans*-C-BM) | 447 | 0.26 |
| 3,4-*cis*-catechin-BM (*cis*-C-BM) | 447 | 0.26 |
| 3,4-*trans*-epicatechin-BM (EC-BM) | 447 | 0.26 |

*Note*: UV-spectra of these flavan-3-ols and their BM adducts are accessible at:

<https://pubs.acs.org/doi/suppl/10.1021/jf103609p/suppl_file/jf103609p_si_002.pdf>

Figure S1. Diagram showing the chemical structures of the benzyl mercaptan adducts from extension subunits and underivatized flavan-3-ols from terminal subunits generated from thiolysis of PCC CT.

**Details of NMR Spectroscopy Acquisition.** ^1^H NMR, ^13^C NMR, and ^1^H−^13^C HSQC NMR spectra were recorded on an Avance 360 (^1^H 360.13 MHz, ^13^C 90.55 MHz) instrument equipped with XWINNMR software (Bruker Corporation, Billerica, MA) or on a BrukerBiospin DMX-500 (1H 500.13 MHz, 13C 125.76 MHz) instrument equipped with Topspin 3.4 software (Bruker Corporation, Billerica, MA). All NMR spectra were recorded in DMSO-*d*6 and were referenced to the residual signals of DMSO-*d*6 (2.49 ppm for ^1^H and 39.5 ppm for ^13^C spectra). For ^1^H−^13^C HSQC experiments on the Avance 360 instrument, spectra were obtained using the standard Bruker pulse program (invietgpsi) with the following parameters: Acquisition: TD 1584 (F2), 768 (F1); SW 11.0 ppm (F2), 160 ppm (F1); O1 1800.65 Hz; O2 7244.38 Hz; D1 = 1.00 s; CNST2 = 145. Acquisition time: F2 channel, 200 ms, F1 channel 2.65 ms. Processing: SI = 1024 (F2, F1), WDW = QSINE, LB = −0.76 Hz (F2), 0.30 Hz (F1); PH_mod = pk; Baseline correction ABSG = 5 (F2, F1), BCFW = 1.00 ppm, BC_mod = quad (F2), no (F1); Linear prediction = no (F2), LPfr (F1). For BrukerBiospin DMX-500 spectra, ^1^H−^13^C HSQC spectra were obtained using the standard Bruker pulse program (hsqcegtpsi) with the following parameters: acquisition, TD 1024 (F2), 256 (F1); SW, 16.0 ppm (F2), 165 ppm (F1); O1, 2350.61 Hz; O2, 9431.83 Hz; D1 = 1.50 s; CNST2 = 145; acquisition time, F2 channel, 6.16 ms; F1 channel, 63.9 ms; processing, SI = 1024 (F2, F1), WDW = QSINE, LB = 1.00 Hz (F2), 0.30 Hz (F1); PH_mod = pk; baseline correction ABSG = 5 (F2, F1), BCFW = 1.00 ppm, BC_mod = quad (F2), no (F1); linear prediction = no (F2), LPfr (F1). Sample sizes used for these spectra ranged from 10 to 15 mg, providing NMR sample solutions with concentrations of 20−30 mg/mL.


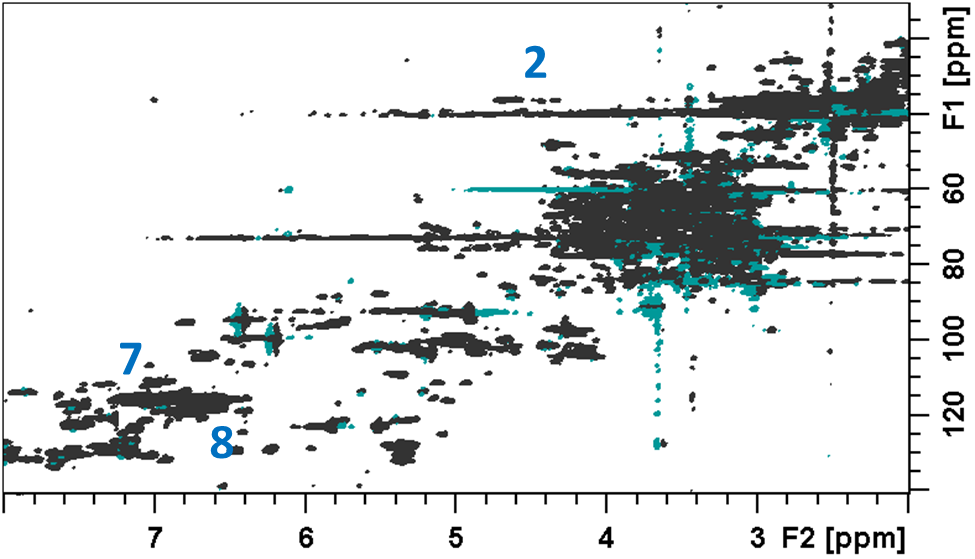


Figure S2. ^1^H-^13^C HSQC NMR spectrum (500/125 MHz) of the acetone/water extract isolated from stems of purple prairie clover (*Dalea purpurea* Vent) at the EF stage. Numbering of cross peak signals coincides with those identified in Figure 3 of the manuscript.


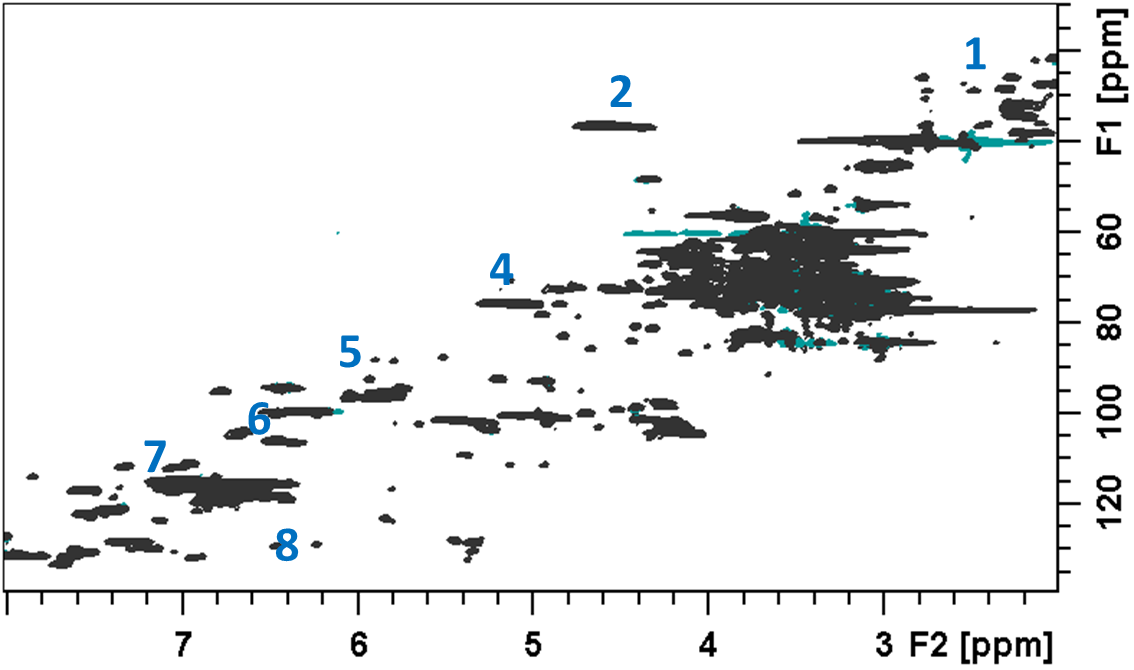


Figure S3. ^1^H-^13^C HSQC NMR spectrum (500/125 MHz) of the acetone/water extract isolated from leaves of purple prairie clover (*Dalea purpurea* Vent) at the EF stage. Numbering of cross peak signals coincides with those identified in Figure 3 of the manuscript.


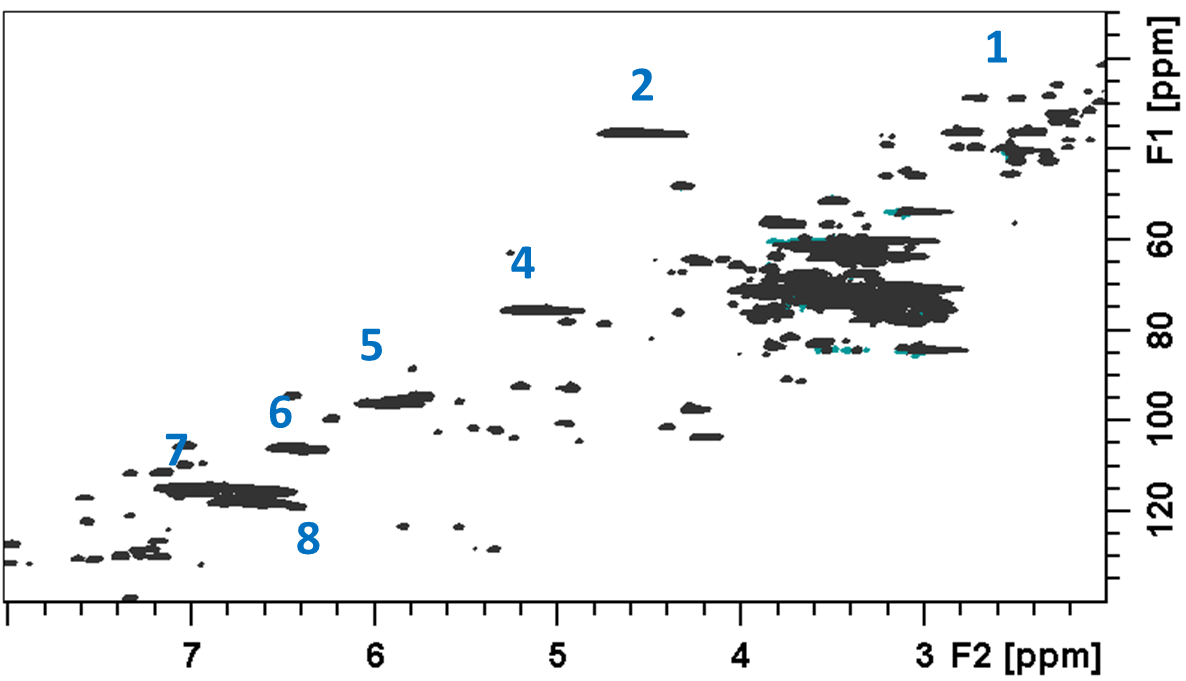


Figure S4. ^1^H-^13^C HSQC NMR spectrum (500/125 MHz) of the acetone/water extract isolated from flowering heads of purple prairie clover (*Dalea purpurea* Vent). Numbering of cross peak signals coincides with those identified in Figure 3 of the manuscript.


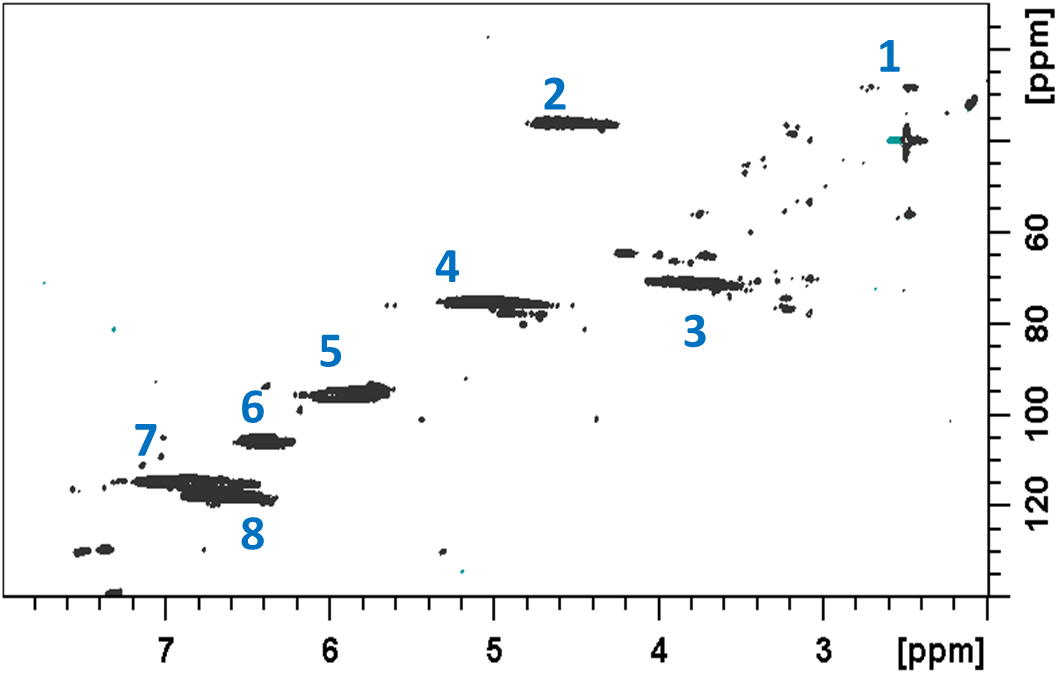


Figure S5. ^1^H-^13^C HSQC NMR spectrum (360/90 MHz) of the F2 Fraction (1:1 acetone/water) isolated from flowering heads of purple prairie clover (*Dalea purpurea* Vent). Numbering of cross peak signals coincides with those identified in Figure 3 of the manuscript.


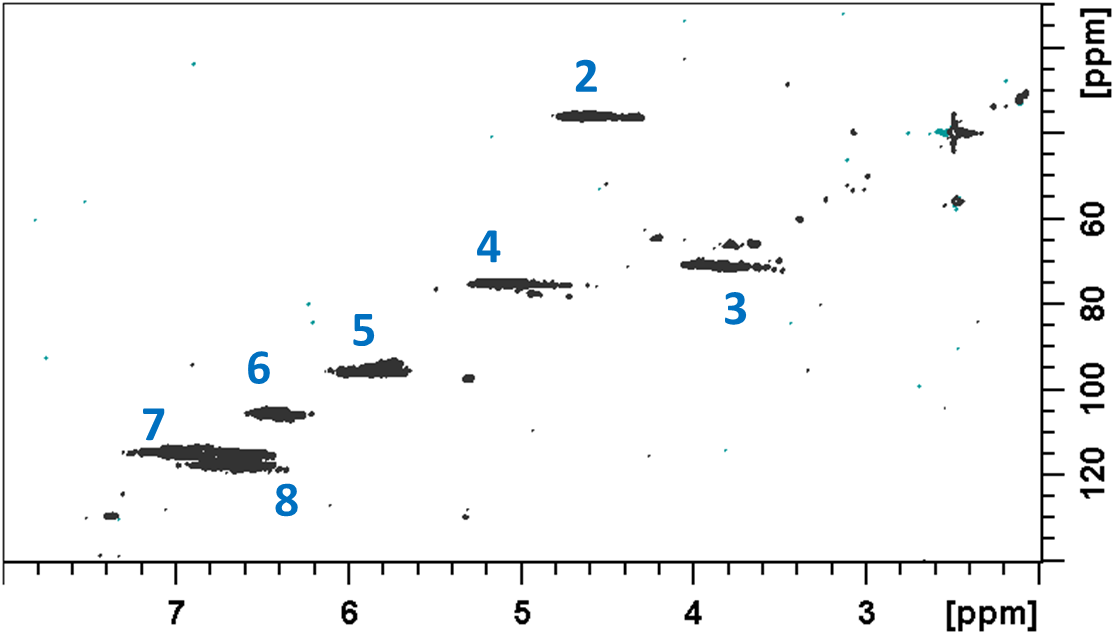


Figure S6. ^1^H-^13^C HSQC NMR spectrum (360/90 MHz) of the F3 Fraction (7:3 acetone/water) isolated from flowering heads of purple prairie clover (*Dalea purpurea* Vent). Numbering of cross peak signals coincides with those identified in Figure 3 of the manuscript.


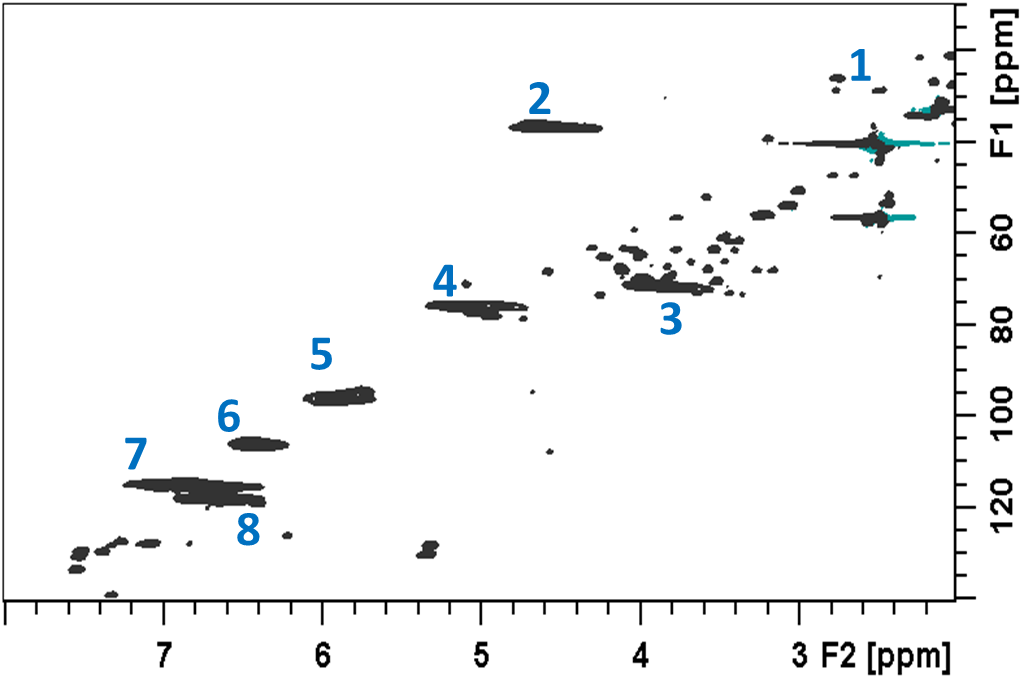


Figure S7. ^1^H-^13^C HSQC NMR spectrum (500/125 MHz) of the F4 Fraction (9:1 acetone/water) isolated from flowering heads of purple prairie clover (*Dalea purpurea* Vent). Numbering of cross peak signals coincides with those identified in Figure 3 of the manuscript.

**Calculating Procyanidin/Prodelphinidin (PC/PD) and cis/trans-Flavan-3-ol Ratios, and mDP.**

The following, with a few modifications, is extracted directly from reference 19 of the

manuscript (Zeller et al *J. Agric. Food Chem*. **2015,** 63, 1967−1973) delineating the method

of determining PC/PD and *cis*/*trans* ratios through integration of cross peaks from the ^1^H-^13^C

HSQC NMR spectra.

The percentage of PCs in the CT sample was calculated using eq 1:

%PC = PC‐6'/[(PD‐2'-6'/2) + PC‐6'] × 100 (1)

where PC-6' is the integration of the contour for the H/C-6' cross-peak of the PC units (label 8, Figure S8) and PD-2'-6' is the integration of the contour for the H/C-2',6' cross-peak of the PD units (label 6, Figure S8). The PD-2'-6' value is divided by 2 to account for the signal arising from two sets of correlated nuclei. The percentage of cis isomers present in the CT sample was calculated through integration of the respective H/C-4 *cis*- and *trans*-flavan-3-ol cross-peak contours (Figure 9S) centered around ^1^H/^13^C chemical shifts of 4.5−4.8/36.0 and 4.4−4.65/37.5 ppm, respectively, and used in eq 2:

% cis flavan-3-ols = cis flavan-3-ols//[cis-flavan-3-ols + trans-flavan-3-ols] x 100 (2)

The following was taken essentially verbatim (with the exception of including the

Figure designation) from the supplemental material of reference 18 of the manuscript

Naumann et al *Molecules* **2018**, 23, 2123.

In mDP estimations, we have found that the terminal H/C-4 cross-peaks integrate for, on average, only about 72% of the other cross-peak signals. Thus, to get the estimate of actual volume of the terminal H/C-4 cross-peaks (Label 1, Figure 10S), we take the observed volume, divide it by 2 (since these signals are arising from two C-H signals) and then divide the resulting value by 0.72. The adjustment can also be made by simply taking the observed volume and multiplying by 0.694. This operation then gives you the corrected terminal H/C-4 cross-peak volume. This value is then divided into the value of the extender H/C-4 cross-peak volume plus the corrected terminal H/C-4 cross-peak volume.

Integrations of cross-peaks were performed in triplicate, and the values

were averaged. Integration of the peaks were performed using Topspin 3.6 software.


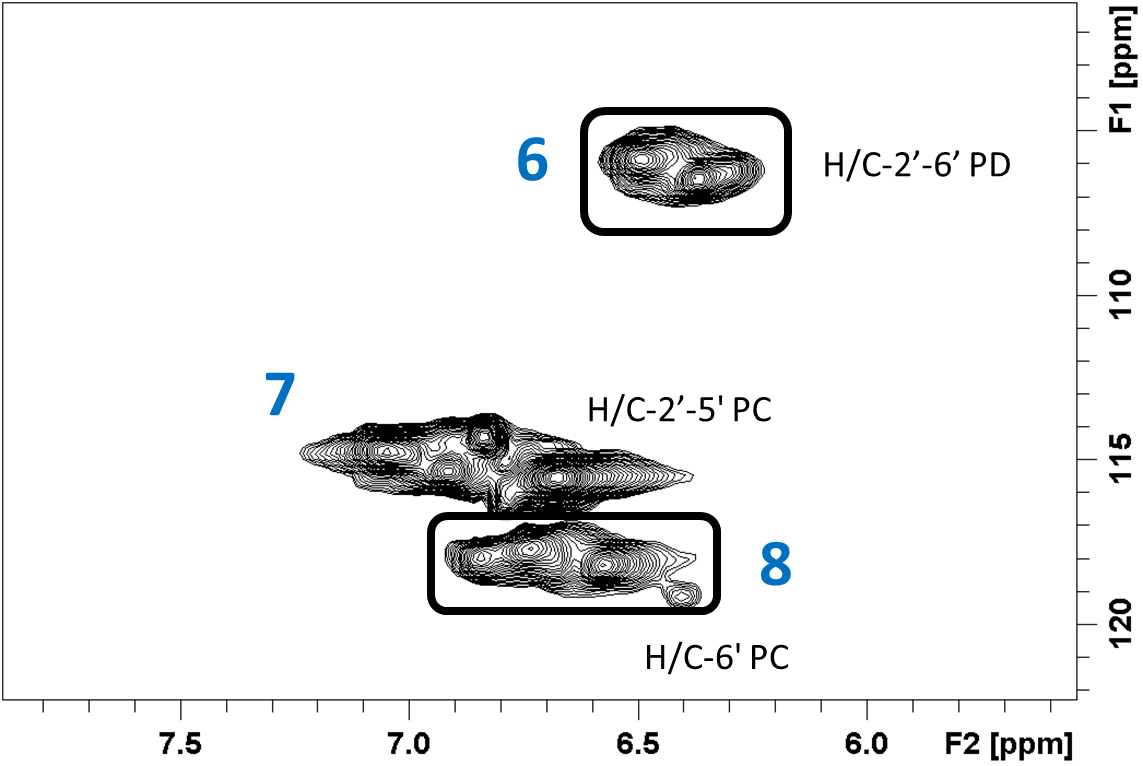


Figure 8S. Diagram showing the NMR cross peaks integrated to provide PC and PD content of CT fractions according to equation 1 above.


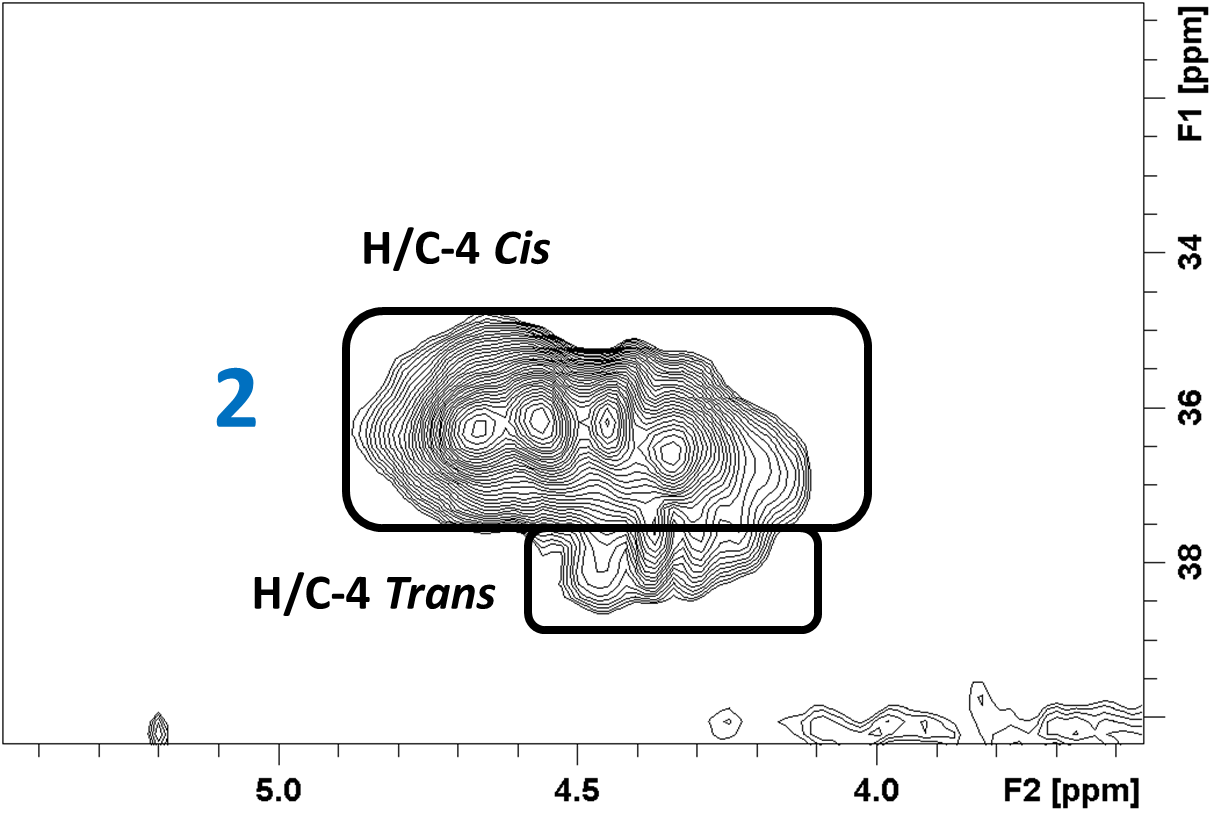


Figure 9S. Diagram showing the NMR cross peaks integrated to provide *cis* and *trans* content of CT fractions according to equation 2 above.


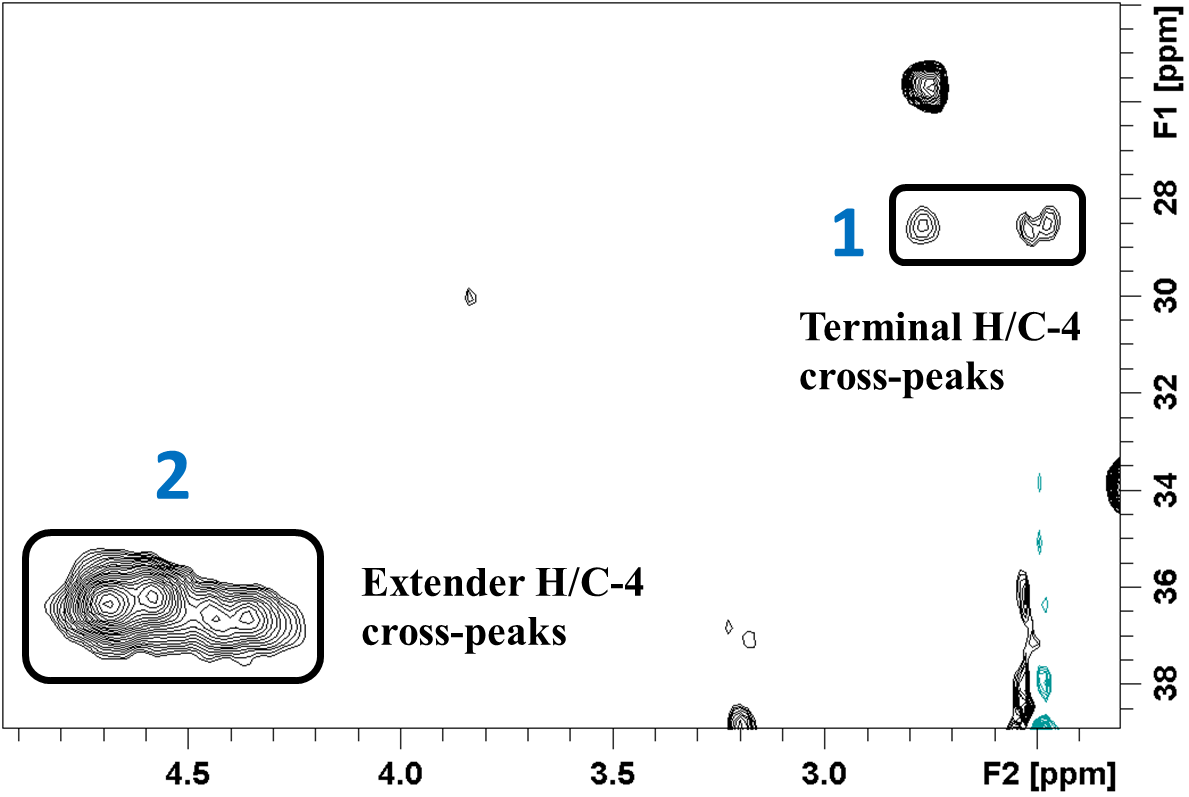


Figure 10S. Diagram showing the NMR cross peaks integrated to provide a calculation of mDP of CT fractions. See discussion of mDP determination given above.

References

Gea, A.; Stringano, E.; Brown, R. H.; Mueller-Harvey, I. In situ analysis and structural elucidation of sainfoin (*Onobrychis viciifolia*) tannins for high-throughput germplasm screening. *J. Agric. Food Chem*. **2011**, 59, 495-503.

Fryganas, C., Drake, C., Ropiak, H.M., Mora-Ortiz, M., Smith, L.M.J., Mueller-Harvey, I., Kowalczyk, R.M. Carbon-13 Cross polarization magic angle spinning nuclear magnetic resonance for measuring proanthocyanidin content and procyanidin to prodelphinidin ratio in sainfoin (*Onobrychis viciifolia*) tissues. *J. Agric. Food Chem.* **2018**, 66, 164073-164081.

Zeller, W.E.; Ramsay, A.; Ropiak, H.M.; Fryganas, C.; Mueller-Harvey, I.; Brown, R.H.; Drake, C.; Grabber, J.H. ^1^H-^13^C HSQC NMR spectroscopy for estimating procyanidin/prodelphinidin and *cis*/*trans* flavanol ratios of condensed tannin fractions: correlation with thiolysis. *J. Agric. Food Chem*. **2015,** 63, 1967−1973.

Naumann, H.; Sepela, R.; Rezaire, A.; Masih, S.E.; Zeller, W.E.; Reinhardt, L.A.; Robe, J.T.; Sullivan, M.L.; Hagerman, A.E. Relationships between structures of condensed tannins from Texas legumes and methane production during in vitro rumen digestion. *Molecules* **2018**, 23, 2123.
